# Risk factors and clinical outcomes for acute kidney injury after surgery for primary spinal and pelvic bone tumours: Analysis of the US Nationwide Inpatient Sample 2005–2020

**DOI:** 10.1016/j.clinme.2026.100651

**Published:** 2026-09-25

**Authors:** Wen Yan Lin, Chun-Yen Chen

**Affiliations:** a Department of Orthopedics, Dalin Tzu Chi Hospital, Buddhist Tzu Chi Medical Foundation, Chiayi, Taiwan; b Department of Orthopedics, China Medical University Hospital, Taichung, Taiwan

**Keywords:** Acute kidney injury (AKI), Nationwide Inpatient Sample (NIS), Pelvic bone tumours, Primary tumour, Spinal tumour, Surgery

## Abstract

**Study objective:**

Primary spinal tumours and pelvic bone tumours are rare but clinically challenging when they arise due to their complex anatomical locations and aggressive behaviour. Surgical resection is first-line treatment but is associated with a high risk of perioperative complications, including acute kidney injury (AKI)—a serious complication with limited evidence in adults undergoing surgery for primary spinal or pelvic bone tumours. This study aimed to evaluate the incidence, risk factors, and outcomes of AKI in adults undergoing surgery for primary spinal or pelvic bone tumours.

**Design:**

Retrospective cohort study.

**Setting:**

Nationwide Inpatient Sample (2006–2020).

**Participants:**

Adults aged 18 and older who underwent surgery for primary spinal or pelvic bone tumours (identified using ICD-9/10 codes).

**Interventions:**

Postoperative AKI (observational study).

**Comparisons:**

AKI.

**Main outcome measure(s):**

Risk factors of postoperative AKI and AKI-associated in-hospital outcomes, including complications, length of stay (LOS), and total hospital charges.

**Results:**

A total of 11,712 inpatients were included, representing 57,164 US hospitalisations after weighting. Postoperative AKI incidence was 2.4%. Patients with AKI had significantly longer hospital stays (adjusted β = 10.60 days), higher total hospital charges (adjusted β = $154,890), and increased risk of acute respiratory failure (aOR = 3.81), venous thromboembolism (aOR = 2.81), sepsis (aOR = 5.11), urinary tract infection (aOR = 2.20), and postoperative transfusion (aOR = 2.72) than those without AKI.

**Conclusions:**

Identifying high-risk patients and implementing perioperative kidney-protective strategies might improve outcomes in adults undergoing surgery for primary spinal or pelvic bone tumours.

## Highlights

• Postoperative AKI occurred in 2.4% after spinal/pelvic tumour surgery.
• AKI increased LOS, costs, and major postoperative complications.
• Renal disease and pelvic tumours were strongest predictors of AKI.
• Advanced age and comorbidities significantly increased AKI risk.
• Early risk stratification may reduce AKI-related adverse outcomes.

## Graphical Abstract

**Figure:**
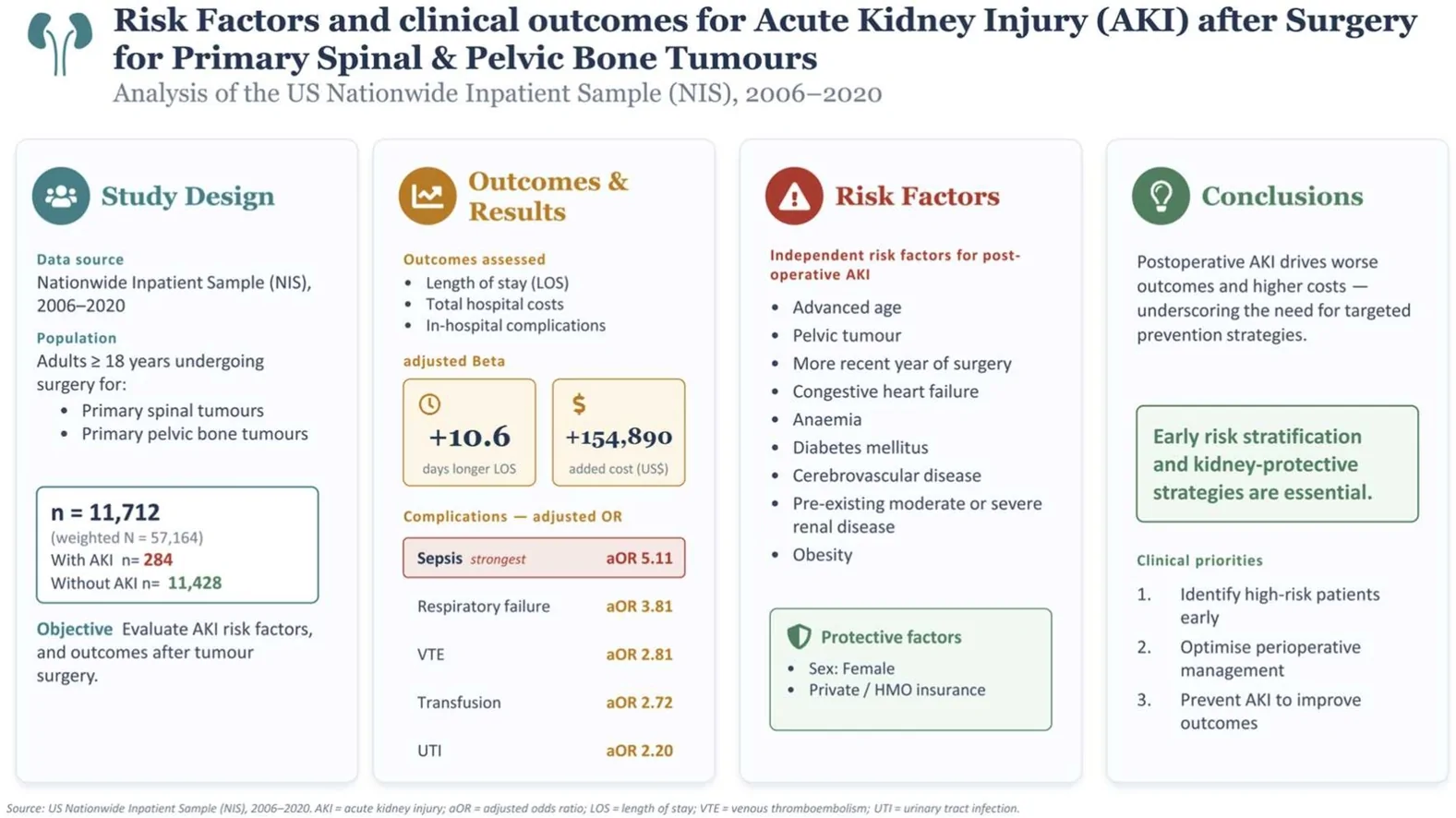

## Introduction

Primary tumours of the spine and pelvis present a significant clinical challenge due to their complex anatomical locations and potential for aggressive biological behaviour.^1^, ^2^ Although these tumours account for only a small fraction of all primary bone malignancies,^3^ they are associated with substantial morbidity because of their proximity to vital neurovascular structures and critical organ systems.^4^ Surgical resection is the foundation of curative treatment for these lesions, particularly in cases of high-grade malignancy or locally aggressive benign tumours.^5^ However, these procedures are typically extensive, technically demanding, and associated with high risk of perioperative complications.^6^

Among various postoperative complications observed in patients undergoing surgery for spinal and pelvic bone tumours, acute kidney injury (AKI) is of particular concern. AKI may arise due to various perioperative factors, including substantial intra- or postoperative blood loss, hypotension, nephrotoxic medication exposure, or prolonged operative time, all of which occur commonly in complex orthopaedic oncologic procedures.^7^ Although AKI is potentially reversible, it is associated with significant short- and long-term consequences, including increased in-hospital mortality, prolonged hospital length of stay, higher healthcare costs, and increased risk of progression to chronic kidney disease (CKD).^8^

Given the serious impact of AKI on patient outcomes, it is essential to identify clinical and procedural factors that predispose patients to this complication. While AKI has been studied extensively in other surgical contexts, such as cardiac^9^ and major abdominal surgery,^10^ limited data are available regarding its incidence and risk factors in spinal and pelvic bone tumour surgery. This study aimed to identify the risk factors for AKI in adults undergoing surgery for primary spinal and pelvic bone tumours.

## Methods

### Study design and data source

This population-based, retrospective, observational study extracted all data from the US Nationwide Inpatient Sample (NIS) database, which is the largest all-payer, continuous inpatient care database in the United States (US), including about eight million hospital stays each year. The database is administered by the Healthcare Cost and Utilization Project (HCUP) of the US National Institutes of Health (NIH). Patient data include primary and secondary diagnoses and procedures, admission and discharge status, patient demographics, expected payment source, hospital length of stay (LOS), and hospital characteristics (i.e., bed size/location/teaching status/hospital region). The continuous, annually updated NIS database derives patient data from about 1,050 hospitals from 44 States in the US, representing a 20% stratified sample of US community hospitals as defined by the American Hospital Association. This study was reported in accordance with the Strengthening the Reporting of Observational Studies in Epidemiology (STROBE) statement. The completed STROBE checklist is provided as a supplementary file.

## Ethics statement

NIS inpatient data were obtained through request to the Online HCUP Central Distributor (available at: https://www.distributor.hcup-us.ahrq.gov/), which administers the database. This study conforms to the NIS data-use agreement with HCUP. Because this study analysed secondary data from the NIS database, patients and the public were not involved directly. Since all data in the NIS database are de-identified, the requirements for Institutional Review Board approval and informed consent were waived.

### Study population

This study included patients aged 18 years and older who underwent surgery for primary spinal or pelvic tumours, as previously described.^11^ Patients were identified using the International Classification of Diseases, Ninth and Tenth Revision (ICD-9 and ICD-10) diagnostic codes encompassing both malignant and benign neoplasms, including tumours of the vertebral column, spinal cord, spinal meninges, pelvic bones, sacrum, and coccyx. For pelvic tumours, the selected diagnostic codes theoretically cover malignant bone sarcomas such as osteosarcoma, chondrosarcoma, and Ewing sarcoma, as well as benign osseous neoplasms; however, histological subtype information was not available in this administrative database and could not be evaluated. Patients with in-hospital mortality, or who had both spinal and pelvic tumours, or lacked data of sex, outcomes, or sampling weights were excluded.

Supplementary Table 1 lists the ICD codes used for case identification.

### Study variables and outcome measures

This study evaluated the incidence of postoperative AKI and in-hospital outcomes, including hospital length of stay (LOS), total hospital costs, and complications. Complications analysed included pneumonia, acute respiratory failure/mechanical ventilation, other respiratory system complications, venous thromboembolism (VTE), periprocedural shock, sepsis, surgical site infection (SSI), urinary tract infection (UTI), postoperative blood transfusion, and organ or vessel perforations.

### Study variables

Patients’ baseline characteristics included age, sex, tumour location, race/ethnicity, household income, insurance status, surgery year, and comorbidities. Comorbidities analysed included coronary artery disease, congestive heart failure (CHF), persistent anaemia, diabetes mellitus (DM), hypertension, cerebrovascular disease, chronic obstructive pulmonary disease (COPD), drug abuse, severe liver disease, pre-existing moderate or severe renal disease, obesity, and Charlson Comorbidity Index (CCI). Hospital-related characteristics such as bed size and location/teaching status were extracted from the database as part of the comprehensive data available for all inpatients.

### Statistical analysis

Descriptive statistics were used to summarise patient demographic and clinical characteristics and outcomes, presenting categorical variables as counts and weighted percentages and continuous variables as means with standard errors (SE). Group comparisons for categorical variables were conducted using the Rao-Scott chi-square test, while weighted mean differences for continuous variables were analysed using survey methods that account for stratification, clustering, and sampling weights, ensuring valid and robust statistical inferences within the context of complex survey designs.

Logistic regression analysis was used to calculate odds ratios (ORs) and 95% confidence intervals (CIs) for binary outcomes, while linear regression analysis was used to estimate coefficients (Beta) and 95% CIs for continuous outcomes. Factors associated with AKI were identified using multivariable logistic regression with backward selection. Candidate variables entered into the initial full model included demographic characteristics, tumour characteristics, individual comorbidities, and hospital characteristics. The AKI odds ratio for age was estimated for each 10-year increase in age. Correlations among candidate variables were examined before model construction to screen for highly correlated variables. The CCI was excluded a priori because its component diseases substantially overlapped with the individual comorbidity variables included in the model. Backward selection was then performed by sequentially removing the variable with the highest p-value and refitting the model until all remaining covariates had p-values <0.05. Multicollinearity was further evaluated using generalised variance inflation factors (GVIFs), with GVIF values <10 considered indicative of no substantial multicollinearity.

All analyses accounted for the complex survey design of the NIS to ensure accurate national estimates. A two-sided p-value of <0.05 was established as statistical significance. All statistical analyses were performed using SAS version 9.4 (SAS Institute Inc., Cary, NC, USA).

## Results

### Study population selection

Fig. 1 summarised the selection process for the study population. A total of 12,152 patients aged ≥18 years who were hospitalised and underwent surgery for primary spinal tumour or pelvic bone tumour were identified in the NIS database from 2006 to 2020 and were included. Patients with in-hospital mortality (n = 79) or had both spinal and pelvic tumours (n = 13) were excluded. Additionally, 348 patients with missing information on sex, total hospital costs, or the weights value of the dataset. After exclusions, 11,712 remaining patients were included, representing 57,164 hospitalisations in the US after weighting (Fig. 1).

**Fig. 1:**
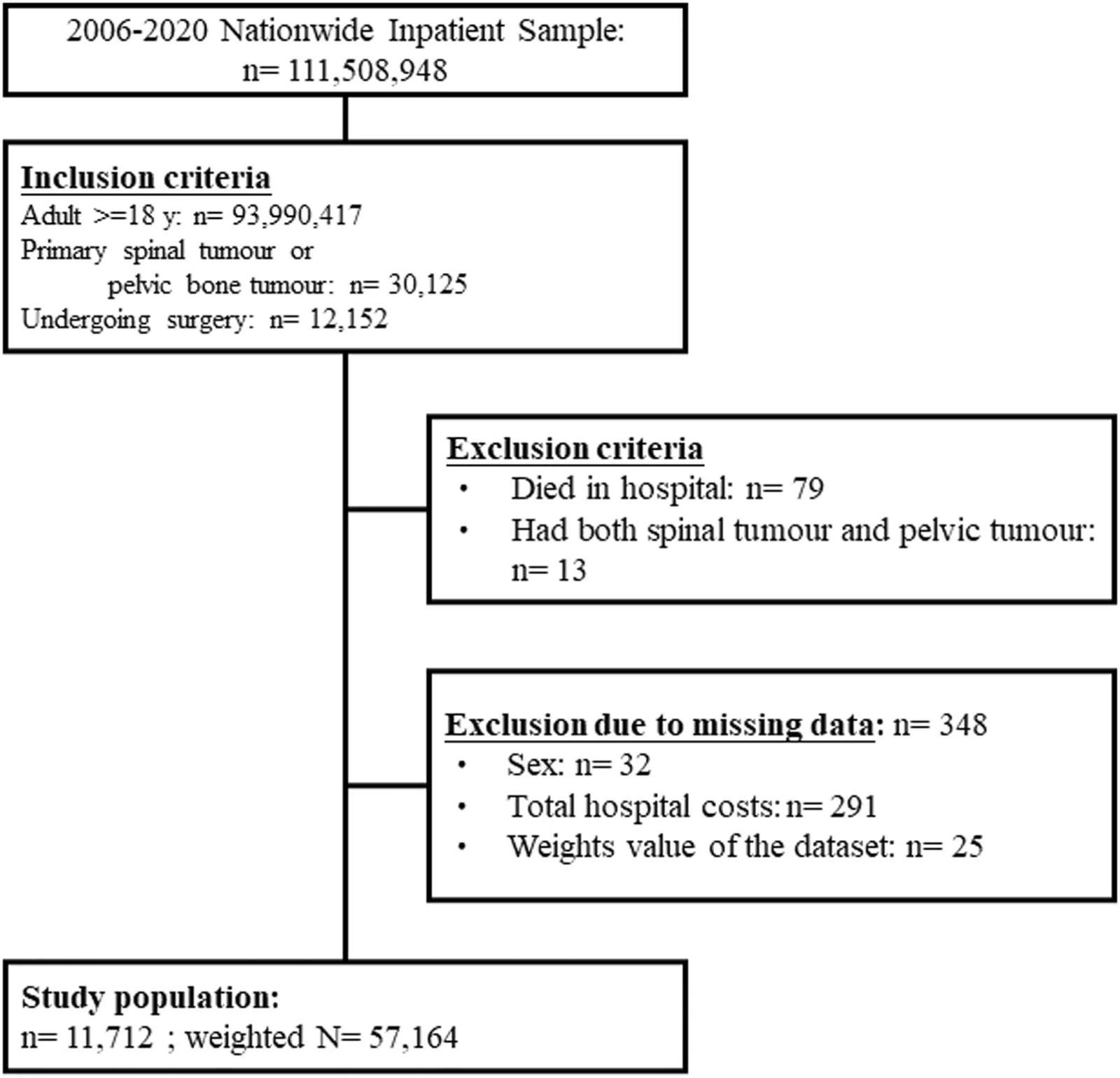
Flow diagram of study cohort selection.

### Characteristics of the study population

Patients’ demographic characteristics (mean age 55.1 years, 54.5% female, 75.0% White), comorbidities, hospital-related information, and outcomes are summarised in Table 1, Table 2. Among all included patients, 90.8% had spinal tumours and 9.2% had pelvic tumours. Regarding surgery year, 43.3% of patients underwent surgery during 2006–2010, 42.1% during 2011–2015, and 14.6% during 2016–2020. Overall, 284 (2.4%) developed AKI postoperatively. The most common comorbidity among these patients was hypertension (44.5%), followed by DM (14.9%) (Table 1).

**Table 1:** Baseline demographic and clinical characteristics of patients with primary spinal or pelvic bone tumours undergoing surgery, stratified by with/without AKI.

	All patients	AKI		*p-value*
	(n = 11,712)	With (n = 284)	Without (n = 11,428)	
**Demography**				
**Age, years**	55.1 ± 0.2	63.5 ± 0.9	54.9 ± 0.2	**<0.001**
18–29	1,053 (9.0)	12 (4.2)	1,041 (9.1)	**<0.001**
30–39	1,246 (10.6)	11 (3.8)	1,235 (10.8)	
40–49	1,814 (15.5)	26 (9.2)	1,788 (15.6)	
50–59	2,562 (21.9)	55 (19.5)	2,507 (22.0)	
60–69	2,544 (21.7)	63 (22.0)	2,481 (21.7)	
70–79	1,737 (14.9)	74 (26.1)	1,663 (14.6)	
80+	756 (6.4)	43 (15.2)	713 (6.2)	
**Sex**				**<0.001**
Male	5,327 (45.5)	171 (60.1)	5,156 (45.1)	
Female	6,385 (54.5)	113 (39.9)	6,272 (54.9)	
**Tumour classification by location**				**<0.001**
Spinal tumour	10,651 (90.8)	202 (70.9)	10,449 (91.3)	
Pelvic tumour	1,061 (9.2)	82 (29.1)	979 (8.7)	
**Surgery year**				**<0.001**
2006–2010	5,154 (43.3)	64 (21.9)	5,090 (43.8)	
2011–2015	4,890 (42.1)	123 (43.5)	4,767 (42.1)	
2016–2020	1,668 (14.6)	97 (34.6)	1,571 (14.1)	
**Race/ethnicity**				0.073
White	7,708 (75.0)	178 (70.3)	7,530 (75.1)	
Black	831 (8.1)	31 (12.1)	800 (8.0)	
Hispanic	885 (8.6)	21 (8.1)	864 (8.6)	
Others	854 (8.3)	24 (9.5)	830 (8.3)	
Missing	1,434	30	1,404	
**Insurance status/Primary payer**				**<0.001**
Medicare/Medicaid	4,960 (42.5)	189 (66.4)	4,771 (41.9)	
Private including HMO	5,896 (50.4)	77 (27.3)	5,819 (51.0)	
Self-pay/no-charge/other	838 (7.1)	18 (6.3)	820 (7.2)	
Missing	18	0	18	
**Household income**				0.101
Q1	2,467 (21.5)	71 (25.7)	2,396 (21.4)	
Q2	2,745 (23.9)	73 (26.5)	2,672 (23.9)	
Q3	2,921 (25.5)	65 (23.8)	2,856 (25.6)	
Q4	3,308 (29.0)	67 (24.1)	3,241 (29.1)	
Missing	271	8	263	
**Comorbidity**				
Coronary artery disease	894 (7.7)	65 (22.9)	829 (7.3)	**<0.001**
CHF	264 (2.3)	41 (14.3)	223 (2.0)	**<0.001**
Persistent anaemia	1,564 (13.4)	104 (36.8)	1,460 (12.8)	**<0.001**
DM	1,740 (14.9)	93 (33.0)	1,647 (14.5)	**<0.001**
Hypertension	5,198 (44.5)	198 (70.0)	5,000 (43.9)	**<0.001**
Cerebrovascular disease	230 (2.0)	24 (8.5)	206 (1.8)	**<0.001**
Chronic pulmonary disease	1,427 (12.2)	53 (18.5)	1,374 (12.0)	**<0.001**
Drug abuse	377 (3.3)	15 (5.4)	362 (3.2)	**0.026**
Severe liver disease	12 (0.1)	1 (0.4)	11 (0.1)	0.192
Pre-existing moderate or severe renal disease	383 (3.3)	87 (30.6)	296 (2.6)	**<0.001**
Obesity	1,365 (11.7)	61 (21.6)	1,304 (11.5)	**<0.001**
**CCI**				**<0.001**
0	6,822 (58.1)	55 (19.4)	6,767 (59.1)	
1	2,046 (17.5)	45 (15.9)	2,001 (17.5)	
2	1,385 (11.8)	56 (19.8)	1,329 (11.6)	
3	496 (4.2)	33 (11.5)	463 (4.1)	
4+	963 (8.3)	95 (33.4)	868 (7.7)	
**Hospital bed size**				0.147
Small	904 (7.5)	24 (8.4)	880 (7.5)	
Medium	2,015 (17.5)	59 (21.0)	1,956 (17.4)	
Large	8,751 (75.0)	200 (70.6)	8,551 (75.1)	
Missing	42	1	41	
**Hospital location/teaching status**				0.357
Rural	223 (1.9)	4 (1.3)	219 (1.9)	
Urban nonteaching	2,338 (19.9)	50 (17.8)	2,288 (20.0)	
Urban teaching	9,109 (78.2)	229 (80.9)	8,880 (78.1)	
Missing	42	1	41	

Abbreviations: AKI, acute kidney injury; HMO, Health Maintenance Organisation; CHF, Congestive heart failure; DM, diabetes mellitus; CCI, Charlson Comorbidity Index.

Continuous variables are presented as mean ± SE; categorical variables are presented as unweighted counts (weighted percentages).

*P-values* <0.05 are shown in bold.

**Table 2:** Outcomes of patients undergoing surgery for primary spinal or pelvic bone tumours, stratified by with/without AKI.

	All patients	AKI		*p-value*
	(n = 11,712)	With (n = 284)	Without (n = 11,428)	
**LOS, days**	7.1 ± 0.1	19.2 ± 1.3	6.8 ± 0.1	**<0.001**
**Total hospital costs, thousand USD**	105.5 ± 1.8	271.3 ± 24.0	101.3 ± 1.6	**<0.001**
**Complication**				
Pneumonia	197 (1.7)	27 (9.5)	170 (1.5)	**<0.001**
Acute respiratory failure/mechanical ventilation	496 (4.2)	59 (20.6)	437 (3.8)	**<0.001**
Other complications of the respiratory system	50 (0.4)	7 (2.4)	43 (0.4)	**<0.001**
VTE	356 (3.1)	39 (13.8)	317 (2.8)	**<0.001**
Periprocedural shock	136 (1.2)	7 (2.6)	129 (1.1)	**0.030**
Sepsis	480 (4.1)	59 (20.4)	421 (3.7)	**<0.001**
SSI	174 (1.5)	20 (7.1)	154 (1.4)	**<0.001**
UTI	818 (7.0)	56 (19.6)	762 (6.7)	**<0.001**
Postoperative blood transfusion	803 (6.9)	72 (25.4)	731 (6.4)	**<0.001**
Perforations of organ or vessels	77 (0.7)	4 (1.5)	73 (0.6)	0.093

Abbreviation: AKI, acute kidney injury; LOS, length of stay; VTE, venous thromboembolism; SSI, surgical site infection; UTI, urinary tract infection.

Continuous variables are presented as mean ± SE; categorical variables are presented as unweighted counts (weighted percentage). *P-values* <0.05 are shown in bold.

Patients with AKI had longer LOS, higher total hospital costs, and higher rates of several complications (acute respiratory failure/mechanical ventilation, VTE, sepsis, UTI, and postoperative blood transfusion (Table 2).

### Associations between AKI (vs. no AKI) and outcomes among patients undergoing surgery for primary spinal or pelvic bone tumours

Table 3 presents the associations between AKI and in-hospital outcomes. After multivariable adjustment, patients with AKI had an estimated mean hospital length of stay that was 10.60 days longer than that of patients without AKI (adjusted beta coefficient [aBeta] = 10.60 days, 95% confidence interval [CI]: 9.85–11.34). They also had an estimated mean total hospital cost that was USD 154,890 higher (aBeta = USD 154,890, 95% CI: USD 143,350–166,430). For binary outcomes, patients with AKI had higher adjusted odds of acute respiratory failure or mechanical ventilation (adjusted odds ratio [aOR] = 3.81, 95% CI: 2.61–5.51), VTE (aOR = 2.81, 95% CI: 1.81–4.31), sepsis (aOR = 5.11, 95% CI: 3.51–7.61), UTI (aOR = 2.20, 95% CI: 1.55–3.11), and postoperative blood transfusion (aOR = 2.72, 95% CI: 1.90–3.90), compared with patients without AKI (Table 3).

**Table 3:** Association between AKI (v.s. no AKI) and outcomes among patients undergoing surgery for primary spinal or pelvic bone tumours.

Outcomes	aBeta/aOR (95% CI)^a^	*p-value*
**LOS**^b^**, days**	10.60 (9.85–11.34)	**<0.001**
**Total hospital charges**^c^**, thousand USD**	154.89 (143.35–166.43)	**<0.001**
**Complication**		
Acute respiratory failure/mechanical ventilation^d^	3.81 (2.61–5.51)	**<0.001**
VTE^e^	2.81 (1.81–4.31)	**<0.001**
Sepsis^f^	5.11 (3.51–7.61)	**<0.001**
UTI^g^	2.20 (1.55–3.11)	**<0.001**
Postoperative blood transfusion^h^	2.72 (1.90–3.90)	**<0.001**

Abbreviation: AKI, acute kidney injury; aBeta, adjusted Beta; aOR, adjusted odds ratio; CI, confidence interval; LOS, length of stay; VTE, venous thromboembolism; UTI, urinary tract infection; CAD, coronary artery disease; CHF, congestive heart failure; DM, diabetes; CVD, cerebrovascular disease; UTI, urinary tract infection; VTE, venous thromboembolism.

*P-values* <0.05 are shown in bold.

a aBeta for continuous outcomes (LOS and Total hospital charges); aOR for binary outcomes.

b Adjusted for related variables of p-value<0.05 in univariate analysis, including age (categorical), sex, race, insurance status, household income, tumour type, hospital bed size, hospital location/teaching status, CAD, CHF, persistent anaemia, DM, CVD, drug abuse, moderate or severe renal disease, and obesity.

c Adjusted for related variables of p-value<0.05 in univariate analysis, including age (continuous), sex, race, insurance status, household income, tumour type, hospital bed size, hospital location/teaching status, CHF, persistent anaemia, DM, CVD, drug abuse, moderate or severe renal disease, and obesity.

d Adjusted for related variables of p-value<0.05 in univariate analysis, including insurance status, tumour type, surgery year of spinal tumour or pelvic bone tumour, hospital bed size, hospital location/teaching status, CAD, CHF, persistent anaemia, DM, hypertension, CVD, chronic pulmonary disease, severe liver disease, moderate or severe renal disease, and obesity.

e Adjusted for related variables of p-value<0.05 in univariate analysis, including age (continuous), sex, insurance status, tumour type, surgery year of spinal tumour or pelvic bone tumour, hospital bed size, hospital location/teaching status, CHF, persistent anaemia, hypertension, CVD, severe liver disease, moderate or severe renal disease, and obesity.

f Adjusted for related variables of p-value<0.05 in univariate analysis, including age (continuous), race, insurance status, tumour type, surgery year of spinal tumour or pelvic bone tumour, hospital location/teaching status, CHF, persistent anaemia, DM, hypertension, CVD, severe liver disease, moderate or severe renal disease, and obesity.

g Adjusted for related variables of p-value<0.05 in univariate analysis, including age (continuous), sex, insurance status, household income, tumour type, hospital location/teaching status, CAD, CHF, persistent anaemia, DM, hypertension, CVD, chronic pulmonary disease, severe liver disease, and moderate or severe renal disease.

h Adjusted for related variables of p-value<0.05 in univariate analysis, including age (categorical), race, insurance status, tumour type, hospital bed size, hospital location/teaching status, CAD, CHF, persistent anaemia, hypertension, CVD, and moderate or severe renal disease.

### Predictors of AKI after surgery for primary spinal and pelvic bone tumours

Table 4 presents the results of regression analyses conducted using backward selection to identify factors associated with AKI. The initial and final backward selection models are provided in Supplementary Table 2. Female patients (aOR = 0.59, 95% CI: 0.46–0.77) and patients covered by private insurance, including HMO (aOR = 0.69, 95% CI: 0.51–0.95), had significantly lower risks of AKI. In contrast, older age (per 10-year increase; aOR = 1.16, 95% CI: 1.06–1.27), pelvic tumours (aOR = 2.87, 95% CI: 2.13–3.88), surgery during 2011–2015 (aOR = 1.81, 95% CI: 1.32–2.49) or 2016–2020 (aOR = 3.01, 95% CI: 2.14–4.23), CHF (aOR = 2.71, 95% CI: 1.66–4.42), persistent anaemia (aOR = 2.56, 95% CI: 1.94–3.37), DM (aOR = 1.67, 95% CI: 1.24–2.24), cerebrovascular disease (aOR = 2.54, 95% CI: 1.46–4.43), moderate or severe renal disease (aOR = 7.21, 95% CI: 5.10–10.18), and obesity (aOR = 1.45, 95% CI: 1.05–1.99) had significantly higher risks of AKI. No substantial multicollinearity was observed (all GVIF values <10) (Table 4).

**Table 4:** Predictors of AKI after surgery for primary spinal and pelvic bone tumours.

Variables	AKI	
	aOR^a^ (95% CI)	*p-value*
**Age, per 10-year increase**^b^	1.16 (1.06–1.27)	**0.002**
**Sex**		
Male	ref.	
Female	0.59 (0.46–0.77)	**<0.001**
**Insurance status/Primary payer**		
Medicare/Medicaid	ref.	
Private including HMO	0.69 (0.51–0.95)	**0.023**
Self-pay/no-charge/other	1.10 (0.66–1.82)	0.713
**Tumour type**		
Spinal tumour	ref.	
Pelvic tumour	2.87 (2.13–3.88)	**<0.001**
**Surgery year**		
2006–2010	ref.	
2011–2015	1.81 (1.32–2.49)	**<0.001**
2016–2020	3.01 (2.14–4.23)	**<0.001**
**Comorbidity**		
Congestive heart failure	2.71 (1.66–4.42)	**<0.001**
Persistent anaemia	2.56 (1.94–3.37)	**<0.001**
Diabetes mellitus	1.67 (1.24–2.24)	**<0.001**
Cerebrovascular disease	2.54 (1.46–4.43)	**<0.001**
Pre-existing moderate or severe renal disease	7.21 (5.10–10.18)	**<0.001**
Obesity	1.45 (1.05–1.99)	**0.025**

Abbreviation: aOR, adjusted odds ratio; AKI, acute kidney injury; CI, confidence interval; HMO, Health Maintenance Organisation; CCI, Charlson Comorbidity Index; Ref., Reference.

*P-values* <0.05 are shown in bold.

a The multivariable model was adjusted using backward selection (*p-value* < 0.05). Candidate variables for backward selection included age, sex, race, primary payer, household income quartile, tumour type, surgery year of spinal tumour or pelvic bone tumour, comorbidity, hospital bed size, and hospital location/teaching status. CCI was excluded because of overlap between its component diseases and the individual comorbidity variables.

b The aOR for age was reported per 10-year increase in age.

## Discussion

Results of the present study show that 2.4% of adults undergoing surgery for primary spinal or pelvic bone tumours developed AKI, and its occurrence was associated with markedly worse clinical outcomes, including longer hospital stays, higher total hospital costs, and increased risk of complications, including acute respiratory failure, VTE, sepsis, UTI, and postoperative blood transfusion, compared to those without AKI. Advanced age, more recent year of surgery, pelvic tumour location, CHF, persistent anaemia, DM, cerebrovascular disease, obesity, and pre-existing moderate to severe renal disease were significant predictors of AKI, while female sex and having private or HMO insurance coverage were protective factors. These findings demonstrate the substantial clinical and economic burden imposed by AKI in adults undergoing surgery for primary spinal or pelvic bone tumours, emphasising the need for effective risk stratification and preventive perioperative management strategies.

The incidence of AKI observed in the present cohort was higher than the rates of 1.1% after lumbar decompression and 1.8% after lumbar fusion reported in a Nationwide Inpatient Sample study of degenerative lumbar surgery,^12^ but was broadly comparable to the 2.96% 90-day incidence reported after multilevel posterior instrumented fusion.^13^ Direct comparison should remain cautious because these studies differed in surgical indications, follow-up periods, case complexity, and methods of AKI ascertainment. The adverse outcome profile in the present study is also consistent with findings from other complex surgical populations. In a prospective multicentre cohort of critically ill neurosurgical patients, postoperative AKI was associated with longer intensive care unit and hospital stays, prolonged mechanical ventilation, higher costs, and increased mortality.^14^ Similarly, persistent AKI following vascular surgery was associated with longer hospitalisation, greater ventilatory support, and higher adjusted costs, even after accounting for comorbidities and other postoperative complications.^15^ These findings support the clinical relevance of the additional hospital stay and costs observed in our cohort. Nevertheless, AKI, respiratory failure, sepsis, transfusion, and other complications may develop from shared perioperative insults or influence one another; therefore, the reported associations should not be interpreted as demonstrating that AKI independently caused the entire increase in resource use. Fluid accumulation, systemic inflammation, and kidney–lung crosstalk may further amplify multiorgan dysfunction after AKI.^8^, ^16^

The risk-factor profile identified in this study was broadly consistent with previous spine-surgery research. In patients undergoing surgery for degenerative lumbar disease, pre-existing chronic kidney disease was the strongest predictor of AKI, while advanced age, congestive heart failure, anaemia, obesity, coagulopathy, and hospital-acquired infections were also associated with increased risk.^12^ Similarly, chronic renal disease, hypertension, diabetes mellitus, advanced age, and congestive heart failure were among the most influential predictors of AKI following multilevel posterior instrumented fusion.^13^ Pre-existing renal disease likely increases susceptibility by reducing functional renal reserve, whereas congestive heart failure may impair effective renal perfusion and increase renal venous congestion. Cerebrovascular disease may additionally indicate systemic vascular vulnerability. Diabetes mellitus and obesity are associated with endothelial dysfunction, microvascular abnormalities, and chronic inflammation, while persistent anaemia may reduce renal oxygen delivery during periods of perioperative blood loss or haemodynamic instability.^8^, ^17^ Although advanced age was independently associated with AKI in the present study, the adjusted odds ratio of 1.01 per year represents a modest annual effect. Age should therefore be considered cumulatively and in conjunction with renal function, comorbidities, and procedural risk, rather than used alone to determine surgical eligibility.

Pelvic tumour location showed one of the strongest associations with AKI in the present analysis. Resection of pelvic and sacral tumours is technically demanding and may involve prolonged operative time, extensive tissue dissection, major haemorrhage, and substantial transfusion requirements. In a series of 137 pelvic tumour resections, 44.5% of patients experienced estimated blood loss exceeding 3,000 mL; acetabular or sacral involvement, larger tumour volume, and a planned operative duration exceeding 200 min independently predicted major blood loss.^18^ These findings provide a plausible explanation for the elevated AKI risk associated with pelvic tumour surgery through hypovolemia, reduced renal perfusion, anaemia, transfusion exposure, and prolonged physiological stress. However, the NIS does not provide operative time, blood loss, intraoperative haemodynamics, nephrotoxic medication exposure, tumour burden, or detailed surgical approach. Pelvic tumour location may therefore partly function as a proxy for unmeasured procedural complexity. Likewise, the lower odds observed among female patients and those with private or health maintenance organisation insurance should be interpreted as epidemiological associations that may reflect differences in baseline health, case selection, healthcare access, or other unmeasured characteristics, rather than direct biological or insurance-mediated protection.

From a clinical perspective, these findings support a structured perioperative kidney-protection pathway for patients undergoing surgery for primary spinal and pelvic bone tumours. Preoperative assessment should integrate baseline kidney function, cardiovascular and cerebrovascular comorbidities, diabetes mellitus, anaemia, obesity, tumour location, and anticipated procedural complexity. For patients considered at increased risk, reasonable measures include medication and nephrotoxin review, optimisation of volume status and haemodynamics, individualised blood-conservation planning, and close postoperative monitoring of serum creatinine and urine output. Current consensus recommendations for noncardiac surgery similarly emphasise early risk identification, avoidance of secondary kidney insults, and kidney health assessment following postoperative AKI.^17^ Future prospective studies should capture kidney disease: improving global outcomes-defined AKI stage and duration, baseline creatinine and urine output, operative time, blood loss, transfusion, hypotension, vasopressor use, nephrotoxic exposure, tumour histology and stage, and surgical approach. External validation of a procedure-specific prediction model, analyses examining whether AKI mediates or marks broader perioperative severity, and evaluation of post-discharge outcomes—including persistent kidney dysfunction, chronic kidney disease, readmission, and mortality—are also warranted. Ultimately, prospective studies or trials evaluating risk-triggered kidney-protective bundles are needed to determine whether these strategies reduce AKI and improve patient outcomes.

### Strengths and limitations

This study has several strengths. First, it used the NIS, a large, nationally representative, all-payer database that captures hospitalisations across different hospital types and regions in the United States. Its large sample size enabled the evaluation of AKI in a rare and clinically complex surgical population. Second, the 15-year study period from 2006 to 2020 allowed temporal variations in patient characteristics and clinical practice to be considered.

Several limitations should be acknowledged. The retrospective observational design precludes causal inference and remains susceptible to selection bias, residual confounding, and misclassification inherent in administrative claims data. AKI was identified using diagnostic codes rather than laboratory-based criteria, and the lack of laboratory and detailed temporal data prevented assessment of AKI stage, severity, and timing. The NIS also lacks granular clinical information, including operative duration, blood loss, haemodynamic instability, transfusion volume, medication and nephrotoxin exposure, surgical approach, and tumour characteristics, limiting adjustment for procedural complexity, disease severity, and other perioperative risk factors.

Patients who died during hospitalisation were excluded because death may truncate the assessment of nonfatal in-hospital outcomes. This may have introduced survivor bias and underestimated the incidence and clinical burden of AKI, although deaths accounted for less than 1% of the initial cohort. Given the small number of deaths, stratification by AKI status was not robust, while the absence of cause-of-death and detailed temporal data prevented assessment of the relationship between AKI and mortality.

The inclusion of heterogeneous spinal and pelvic tumours may obscure differences across tumour subtypes and surgical procedures. In addition, the predominance of spinal tumours and White patients limits the generalisability of subgroup findings to pelvic tumours and other racial and ethnic groups. Finally, because the NIS captures individual hospitalisations without longitudinal follow-up, post-discharge kidney recovery, readmission, chronic kidney disease progression, and long-term mortality could not be assessed.

## Conclusions

AKI is a serious postoperative complication among patients undergoing surgery for primary spinal and pelvic bone tumours, increasing hospital length of stay, healthcare costs, and risk of serious complications. Independent factors associated with AKI in these patients include pelvic tumour location, advanced age, and multiple comorbid conditions such as heart failure, DM, and pre-existing renal disease. Female sex and private insurance are associated with lower risk. Results of the present study may inform risk stratification, kidney-protective strategies and perioperative management strategies to improve outcomes in high-risk patients undergoing surgery for primary spinal and pelvic bone tumours.

## CRediT authorship contribution statement

**Wen Yan Lin:** Writing – review & editing, Writing – original draft, Validation, Software, Project administration, Investigation, Formal analysis, Conceptualization. **Chun-Yen Chen:** Visualization, Supervision, Resources, Methodology, Funding acquisition, Data curation.

## Ethics approval and consent to participate

NIS inpatient data were obtained through request to the Online HCUP Central Distributor (available at: https://www.distributor.hcup-us.ahrq.gov/), which administers the database. This study conforms to the NIS data-use agreement with HCUP. Because this study analysed secondary data from the NIS database, patients and the public were not involved directly. Since all data in the NIS database are de-identified, the requirements for Institutional Review Board approval and informed consent were waived.

## Funding

This research did not receive any specific grant from funding agencies in the public, commercial, or not-for-profit sectors.

## Declaration of competing interest

The authors declare that they have no known competing financial interests or personal relationships that could have appeared to influence the work reported in this paper.

## Data Availability

The original contributions presented in this study are included in the article/supplementary material. Further inquiries can be directed to the corresponding author.

## References

[1] Ariyaratne S, Jenko N., Iyengar K.P., James S., Mehta J., Botchu R. (2023). Primary osseous malignancies of the spine. Diagnostics.

[2] Soares do Brito J., Esperança-Martins M., Abrunhosa-Branquinho A. (2022). Management of unresectable localized pelvic bone sarcomas: current practice and future perspectives. Cancers.

[3] Oitment C., Bozzo A., Martin A.R. (2021). Primary sarcomas of the spine: population-based demographic and survival data in 107 spinal sarcomas over a 23-year period in Ontario, Canada. Spine J.

[4] Laitinen M.K., Parry M.C., Morris G.V., Jeys L.M. (2023). Pelvic bone sarcomas, prognostic factors, and treatment: a narrative review of the literature. Scand J Surg.

[5] Charest-Morin R., Fisher C.G., Sahgal A. (2019). Primary bone tumor of the spine-an evolving field: what a general spine surgeon should know. Global Spine J.

[6] Feler J., Sun F., Bajaj A. (2022). Complication avoidance in surgical management of vertebral column tumors. Curr Oncol.

[7] Tahir M., Nawaz H., Iqbal A. (2025). Incidence and clinical outcomes of postoperative acute kidney injury in relation to etiological factors and surgical procedures. Cureus.

[8] Ostermann M., Lumlertgul N., Jeong R., See E., Joannidis M., James M. (2025). Acute kidney injury. Lancet.

[9] Cheruku S.R., Raphael J., Neyra J.A., Fox A.A. (2023). Acute kidney injury after cardiac surgery: prediction, prevention, and management. Anesthesiology.

[10] Mikkelsen T.B., Schack A., Oreskov J.O., Gögenur I., Burcharth J., Ekeloef S. (2022). Acute kidney injury following major emergency abdominal surgery - a retrospective cohort study based on medical records data. BMC Nephrol.

[11] Ahmed A.K., Goodwin C.R., De la Garza-Ramos R. (2017). Predicting short-term outcome after surgery for primary spinal tumors based on patient frailty. World Neurosurg.

[12] Hsieh Y.Y., Wu L.C., Chen I.C., Chiang C.J. (2024). Incidence and predictors of acute kidney injury after elective surgery for lumbar degenerative disease: a 13-year analysis of the US Nationwide Inpatient Sample. J Chin Med Assoc.

[13] Heo K.Y., Rajan P.V., Khawaja S., Barber L.A., Yoon S.T. (2024). Machine learning approach to predict acute kidney injury among patients undergoing multi-level spinal posterior instrumented fusion. J Spine Surg.

[14] Deng Y., Yuan J., Chi R. (2017). The incidence, risk factors and outcomes of postoperative acute kidney injury in neurosurgical critically ill patients. Sci Rep.

[15] Filiberto A.C., Adiyeke E., Ozrazgat-Baslanti T. (2024). Persistent acute kidney injury is associated with poor outcomes and increased hospital cost in vascular surgery. Ann Vasc Surg.

[16] Herrlich A. (2022). Interorgan crosstalk mechanisms in disease: the case of acute kidney injury-induced remote lung injury. FEBS Lett.

[17] Prowle J.R., Forni L.G., Bell M. (2021). Postoperative acute kidney injury in adult non-cardiac surgery: joint consensus report of the Acute Disease Quality Initiative and PeriOperative Quality Initiative. Nat Rev Nephrol.

[18] Tang X., Guo W., Yang R., Tang S., Ji T. (2009). Evaluation of blood loss during limb salvage surgery for pelvic tumours. Int Orthop.

