# Supplementary material for: Risk factors and clinical outcomes for acute kidney injury after surgery for primary spinal and pelvic bone tumours: Analysis of the US Nationwide Inpatient Sample 2005–2020
Source: Clin Med (Lond). 2026 Sep 25;26(6):100651. doi: 10.1016/j.clinme.2026.100651 (PMC13673342; doi:10.1016/j.clinme.2026.100651)
Supplement: Supplementary file 1 [file mmc1.docx]

Supplementary Table S1. ICD codes used to define diagnosis and procedures.

|  | ICD-9 | ICD-10 |
| --- | --- | --- |
| Spinal tumour | 170.2, 192.2, 192.3, 213.2, 225.3, 225.4, 237.6 | C41.2, C70.1, C72.0, D16.6, D32.1, D33.4, D42.1 |
| Pelvic bone tumour | 170.6, 213.6 | C41.4, D16.8 |
| Surgery | ICD-9-PCS: 79, 80, 81, 03, 01 | ICD-10-PCS: 0QS, 0PS, 0QB, 0PB, 0RP, 0SP, 0SB, 0SG, 0RW, 0RG, 0RN |
| Acute Kidney Injury | 584 | N17 |
| Coronary artery disease | 410-414 | I25, I20-I24 |
| Congestive heart failure |  | I09.9, I11.0, I13.0, I13.2, I25.5, I42.0, I42.5-I42.9, I43, I50, P29.0 |
| Persistent anaemia | 285 | D63, D64 |
| Diabetes |  | E10-E14 |
| Hypertension | 401-405 | I10-I15 |
| Cerebrovascular disease | 362.34, 430-438 | G45, G46, H34.0, I60-I69 |
| Chronic pulmonary disease | 416.8, 416.9, 490-505, 506.4, 508.1, 508.8 | I27.8, I27.9, J40-J47, J60-J67, J68.4, J70.1, J70.3 |
| Drug abuse |  | Z71.5, F10-F19 |
| Severe liver disease | 456.0-456.2, 572.2-572.8 | I85.0, I85.9, I86.4, I98.2, K70.4, K71.1, K72.1, K72.9, K76.5, K76.6, K76.7 |
| Moderate or severe renal disease | 403.01, 403.11, 403.91, 404.02, 404.03, 404.12, 404.13, 404.92, 404.93, 582.x, 583.0-583.7, 585, 586, 588.0, V42.0, V45.1, V56 | I12.0, I13.1, N03.2-N03.7, N05.2-N05.7, N18, N19, N25.0, Z49.0-Z49.2, Z94.0, Z99.2 |
| Obesity | 278.00, 278.01, V85.3, V85.4 | E66.01, E66.09, E66.1, E66.2, E66.8, E66.9, Z68.3, Z68.4 |
| Pneumonia | 486, 481, 482.8, 482.3 | A48.1, J12 J13, J14, J15, J16, J17, J18, J69.0, J95.4, J95.851 |
| Acute respiratory failure/ Mechanical ventilation | 518.5, 518.81-518.84 ICD-9-PCS:96.7 | J95.1, J95.82, Z99.12 ICD-10-PCS: 5A1935Z, 5A1945Z, 5A1955Z |
| Other complications of respiratory system | 997.32, 997.39, 519.0 | J95.88, J95.89, J95.0, J98.0 |
| Venous thromboembolism | 415, 451-453, 671, 673, 997.2 | I260, I269, I801-803, I808, I809, I820-I823, I828, I829, O082, O223, O871, O882, I81, I82 |
| Periprocedural shock, hypertension, or other cardiovascular complications | 998.00, V58.89, 909.3, 997.91, 997.1, 997.2, 997.7 | T81.1, I97.3, I97.88, T81.7 |
| Sepsis | 995.9, 996.64, 038, 999.3, 790.7, 041, 785.52 | R78.81, A41, R65.2, T80.2, A42.7, A22.7, B37.7, A26.7, A28.2, A54.86, B00.7, A32.7, A24.1, A39.2, A20.7, A21.7, A48.3 |
| Surgical Site Infection | 998.31, 998.32, 998.5 | T81.31XA, T81.4 |
| Urinary Tract Infection | 590, 595, 597, 599.0, 601 | N10, N30.0, N30.9, N34.1, N39.0, N39.8, N39.9 |
| Postoperative blood transfusion | ICD-9-PCS: 99.04 | ICD-10-PCS: 30233N1 |
| Perforations of organ or vessels | 998.2 | D78.12, G97.49, E36.12, K91.71, N99.72, T88.8XXA, M96.821, L76.12 |

**Supplementary Table 2. Initial and final backward selection models for risk factors of AKI**

|  | **Initial full model** | | **Final model** | |
| --- | --- | --- | --- | --- |
|  | **aOR (95% CI)** | ***p-value*** | **aOR (95% CI)** | ***p-value*** |
| **Demography** |  |  |  |  |
| **Age, per 10-year increase ^a^** | 1.11 (1.001-1.24) | **0.047** | 1.16 (1.06-1.27) | **0.002** |
| **Sex** |  |  |  |  |
| Male | Ref. | **-** | Ref. | **-** |
| Female | 0.56 (0.43-0.74) | **<0.001** | 0.59 (0.46-0.77) | **<0.001** |
| **Tumour classification by location** |  |  |  |  |
| Spinal tumour | Ref. | **-** | Ref. | **-** |
| Pelvic tumour | 3.05 (2.23-4.17) | **<0.001** | 2.87 (2.13-3.88) | **<0.001** |
| **Surgery year** |  |  |  |  |
| 2006-2010 | Ref. | **-** | Ref. | **-** |
| 2011-2015 | 1.76 (1.24-2.49) | **0.001** | 1.81 (1.32-2.49) | **<0.001** |
| 2016-2020 | 3.07 (2.12-4.46) | **<0.001** | 3.01 (2.14-4.23) | **<0.001** |
| **Race/ethnicity** |  |  |  |  |
| White | Ref. | **-** |  |  |
| Black | 1.54 (1.00-2.38) | 0.051 |  |  |
| Hispanic | 1.11 (0.69-1.81) | 0.666 |  |  |
| Others | 1.32 (0.87-2.01) | 0.186 |  |  |
| **Insurance status/Primary payer** |  |  |  |  |
| Medicare/Medicaid | Ref. | **-** | Ref. | **-** |
| Private including HMO | 0.71 (0.50-0.99) | **0.044** | 0.69 (0.51-0.95) | **0.023** |
| Self-pay/no-charge/other | 0.97 (0.55-1.72) | 0.926 | 1.10 (0.66-1.82) | 0.713 |
| **Household income** |  |  |  |  |
| Q1 | Ref. | **-** |  |  |
| Q2 | 0.92 (0.64-1.32) | 0.644 |  |  |
| Q3 | 0.79 (0.55-1.14) | 0.202 |  |  |
| Q4 | 0.74 (0.51-1.08) | 0.120 |  |  |
| **Comorbidity** |  |  |  |  |
| Coronary artery disease | 1.32 (0.89-1.95) | 0.161 |  |  |
| CHF | 2.16 (1.22-3.80) | **0.008** | 2.71 (1.66-4.42) | **<0.001** |
| Persistent anaemia | 2.58 (1.91-3.49) | **<0.001** | 2.56 (1.94-3.37) | **<0.001** |
| DM | 1.59 (1.14-2.21) | **0.006** | 1.67 (1.24-2.24) | **<0.001** |
| Hypertension | 1.06 (0.75-1.48) | 0.742 |  |  |
| Cerebrovascular disease | 2.97 (1.66-5.30) | **<0.001** | 2.54 (1.46-4.43) | **<0.001** |
| Chronic pulmonary disease | 0.98 (0.65-1.46) | 0.907 |  |  |
| Drug abuse | 0.85 (0.46-1.55) | 0.585 |  |  |
| Severe liver disease | 1.50 (0.13-17.29) | 0.747 |  |  |
| Moderate or severe renal disease | 6.88 (4.76-9.95) | **<0.001** | 7.21 (5.10-10.18) | **<0.001** |
| Obesity | 1.46 (1.04-2.06) | **0.029** | 1.45 (1.05-1.99) | **0.025** |
| **Hospital bed size** |  |  |  |  |
| Small | Ref. | **-** |  |  |
| Medium | 1.07 (0.65-1.77) | 0.790 |  |  |
| Large | 0.90 (0.58-1.40) | 0.630 |  |  |
| **Hospital location/teaching status** |  |  |  |  |
| Rural | Ref. | **-** |  |  |
| Urban nonteaching | 1.91 (0.72-5.04) | 0.190 |  |  |
| Urban teaching | 1.75 (0.68-4.49) | 0.242 |  |  |

Abbreviations: AKI, acute kidney injury; aOR, adjusted odds ratio; HMO, Health Maintenance Organisation; CHF, Congestive heart failure; DM, diabetes mellitus; CCI, Charlson Comorbidity Index; Ref., Reference.

Age (category) was not simultaneously included because it represents the same underlying variable as age.

CCI was not entered into the backward selection model because its component diseases overlapped with the individual comorbidity variables included in the analysis.

^a^ The aOR for age was reported per 10-year increase in age.
